# Implementation of WHO multimodal strategy for improvement of hand hygiene: a quasi-experimental study in a Traditional Chinese Medicine hospital in Xi’an, China

**DOI:** 10.1186/s13756-017-0254-4

**Published:** 2017-09-20

**Authors:** Li Shen, Xiaoqing Wang, Junming An, Jialu An, Ning Zhou, Lu Sun, Hong Chen, Lin Feng, Jing Han, Xiaorong Liu

**Affiliations:** 1 Department of Infection Control, Xi’an Hospital of Traditional Chinese Medicine, No.69 Feng Cheng 8th Road, Weiyang District, Xi’an, 710021 China; 2 Department of Acupuncture and Moxibustion, Xi’an Hospital of Traditional Chinese Medicine, No.69 Feng Cheng 8th Road, Weiyang District, Xi’an, 710021 China; 3 Department of Information Consultation, Library of Xi’an Jiaotong University, No.76 Yan Ta West Road, Yanta District, Xi’an, 710061 China; 4 Department of Cadre Health Care, Xi’an Hospital of Traditional Chinese Medicine, No.69 Feng Cheng 8th Road, Weiyang District, Xi’an, 710021 China

**Keywords:** Hand hygiene, Compliance, Correctness, Healthcare-associated infection

## Abstract

**Background:**

Hand hygiene (HH) is an essential component for preventing and controlling of healthcare-associated infection (HAI), whereas compliance with HH among health care workers (HCWs) is frequently poor. This study aimed to assess compliance and correctness with HH before and after the implementation of a multimodal HH improvement strategy launched by the World Health Organization (WHO).

**Methods:**

A quasi-experimental study design including questionnaire survey generalizing possible factors affecting HH behaviors of HCWs and direct observation method was used to evaluate the effectiveness of WHO multimodal HH strategy in a hospital of Traditional Chinese Medicine. Multimodal HH improvement strategy was drawn up according to the results of questionnaire survey. Compliance and correctness with HH among HCWs were compared before and after intervention. Also HH practices for different indications based on WHO “My Five Moments for Hand Hygiene” were recorded.

**Results:**

In total, 553 HCWs participated in the questionnaire survey and multimodal HH improvement strategy was developed based on individual, environment and management levels. A total of 5044 observations in 23 wards were recorded in this investigation. The rate of compliance and correctness with HH improved from 66.27% and 47.75% at baseline to 80.53% and 88.35% after intervention. Doctors seemed to have better compliance with HH after intervention (84.04%) than nurses and other HCWs (81.07% and 69.42%, respectively). When stratified by indication, compliance with HH improved for all indications after intervention (*P* < 0.05) except for “after body fluid exposure risk” and “after touching patient surroundings”.

**Conclusion:**

Implementing the WHO multimodal HH strategy can significantly improve HH compliance and correctness among HCWs.

**Electronic supplementary material:**

The online version of this article (10.1186/s13756-017-0254-4) contains supplementary material, which is available to authorized users.

## Background

Healthcare-associated infection (HAI) represents a major burden and safety issue for patients in the developing countries, with severe and greatly underestimated effect on patients and health care systems [1]. According to the survey of National HAI Surveillance System, in 2014, at least 26,972 cases of HAI arose in patients admitted to hospital in China [2]. HAI resulted in prolonged length of hospital stay, direct economic loss, morbidity and mortality among hospitalized patients [3]. A recent study in China identified that the average cost of hospitalization increased ¥13,839.16(€1792.64) due to HAI [4]. The hands of healthcare workers (HCWs) can be a major mode of transmission of microbial pathogens by touching the environment or patients’ skin during healthcare delivery, which supports that hand hygiene (HH) is a critical component of a bundle approaches for preventing and controlling HAIs [5–9]. The World Health Organization (WHO) launched a multimodal strategy in 2009 to improve HH practice worldwide, which includes 5 important components: (1) system change, (2) training and education, (3) evaluation and feedback, (4) reminders in the workplace (5) institutional safety climate [10]. It has been demonstrated the implementation of WHO HH strategy is feasible and effective to enhance hand hygiene compliance, which leads to a reduction of HAI [11–14]. However, there have been few data on the implementation of the WHO multimodal HH strategy in China. We initiated this study of implementation of WHO multimodal HH strategy in order to improve awareness of HAI and enhance HH compliance and correctness among HCWs.

## Methods

The study was conducted in Xi’an Hospital of Traditional Chinese Medicine (TCM), Xi’an, China, between September 2015 and August 2016. It is the largest public hospital in north Xi’an, which is the capital city of Shaanxi Province. This hospital has 1001 beds in 27 clinical departments including acupuncture and moxibustion, intensive care, emergency, surgical and TCM subspecialties with 1377 HCWs. We performed this two-part quasi-experimental study including questionnaire survey of factors affecting HH behaviors of HCWs and direct observation of compliance and correctness with HH before and after intervention.

### Part I: Questionnaire survey

In this part, we did a questionnaire survey on possible factors affecting HH behaviors of HCWs. Each participant voted those factors contributing to HH noncompliance from the questionnaire. On the basis of the reasons for HH noncompliance summarized in the questionnaire, multimodal improvement strategy was developed accordingly.

### Part II: Observation of compliance and correctness before and after intervention

In this part, detailed intervention measures were drawn up and then implemented according to the multimodal improvement strategy acquired from the results of questionnaire survey. We collected observational data on compliance and correctness with HH before and after intervention respectively.

Observation sessions were performed by 9 trained student nurses. The training course included HH indications and correct HH techniques recommended by WHO. A standard form was used to record the HH compliance and correctness. Observers were taught how to complete the form and record the number of HH actions and HH opportunities. We defined an opportunity as the occurrence of any indication during the observed care sequences. We recorded actions, either handwashing or hand rubbing based on WHO “My Five Moments for Hand Hygiene”: before touching a patient, before clean/aseptic procedure, after body fluid exposure risk, after touching a patient, and after touching patient surroundings [15]. Since an indication for HH was related to the risk of pathogen transmission from one surface to another, we added two more WHO recommended indications in our study: if moving from a contaminated body site to a clean body site during patient care, after removing gloves [16]. An action with correct HH techniques must satisfy three criteria: (1) rub hands with 6-step HH techniques; (2) duration of the rub procedure lasts 15 s at least; (3) dry hands with disposable paper towels. Each observer monitored the HH practice of HCWs for 45–60 min.

Compliance and correctness with HH were compared before and after the implementation. HCWs including doctors, nurses, technicians, interns and cleaners were observed for HH actions and HH opportunities. Data of technicians, interns and cleaners was combined as other HCWs. We expressed HH compliance as the proportion of predefined opportunities met by HH actions. And HH correctness was regarded as the proportion of all HH actions met by HH actions with correct techniques. All the data was analyzed with SPSS version 16.0. The Chi square test was applied to test the statistical difference in HH compliance and correctness before and after the implementation. Also HH compliance stratified by professional category and indication was calculated. Results with *P* < 0.05 were considered statistically significant.

## Results

### Part I: Questionnaire survey

**Fig. 1:**
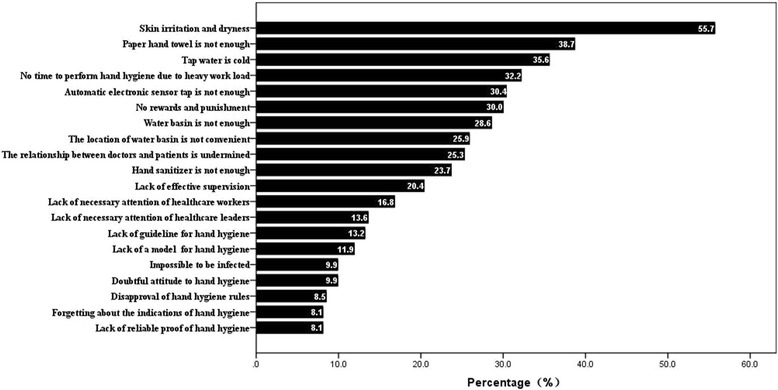
Possible factors affecting HH behaviors of HCWs in the questionnaire survey

**Fig. 2:**
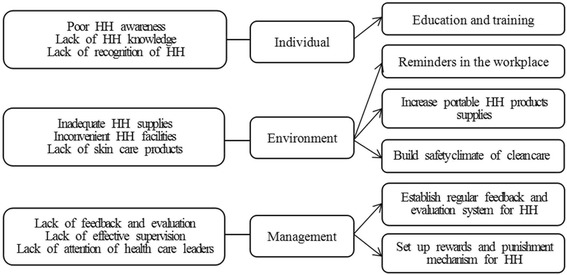
Main reasons for HH noncompliance and corresponding improvement measures

A total of 558 HCWs from 37 departments participated in this survey. Of these, 553 (99.10%) completed the baseline questionnaire. The general information of all participants was summarized in Table 1. Each participant voted those factors contributing to HH noncompliance from the questionnaire. All the possible factors affecting HH behaviors of HCWs were arranged in descending order according to the number of votes (Fig. 1). The main reasons for HH noncompliance were classified into individual, environment and management levels. Multimodal improvement strategy was drafted accordingly (Fig. 2) and detailed intervention measures were drawn up at the same time.

**Table 1:** The general information of 553 participants in the survey

		N (cases)	Percentage (%)	Mean (±s)
Age		553		31.78 ± 8.120
Gender	Female	433	78.30	
	Male	120	21.70	
Work experience	<1 year	104	18.81	
	1 ~ 5 years	139	25.13	
	5 ~ 10 years	155	28.03	
	≥10 years	155	28.03	
Education level	Senior high school	14	2.53	
	Junior college	181	32.73	
	College	273	49.37	
	Post graduate	85	15.37	
Profession	Doctors	191	34.54	
	Nurses	300	54.25	
	Technicians	35	6.33	
	Interns	23	4.16	
	Cleaners	4	0.72

For the individual reasons such as poor HH awareness, full training campaign on HH techniques among HCWs was carried out. Our management of infection control department first participated in the training of WHO “My Five Moments for Hand Hygiene” provided by Xi’an Quality Control Center of Nosocomial Infection. Then we shoot instructional videos on five key moments for HH and correct HH techniques in our hospital wards with our HCWs. After that they were called together to study HH knowledge via videos and PPT (based on WHO training slides). All the HCWs including doctors, nurses, interns, student nurses, lab technicians and cleaners should attend educational courses on HH every year. A posttest format was used to assess training efficacy after each course.

Inadequate HH supplies and inconvenient HH facilities was another cause for noncompliance with HH in our hospital. We took a series of measures to improve HH facilities: increasing supplies of pocket alcohol-based hand rub (ABHR) and disposable paper towels; making sure every wash basin equipped with disposable paper towels and poster for correct handwashing techniques; replacing water tap in nurse station with automatic electronic sensor tap; distributing skin care products to HCWs. Colorful HH posters were placed in the doctor’s office in each ward with WHO “My Five Moments for Hand Hygiene”. A little tip for HH was placed at the edge of the computer screen of nurse station. Visible reminders for HH were also set at the entrance of each ward. To create a better environment for HH, we offered large-scale HH improvement campaign to both HCWs and patients with knowledge contest, visible display boards, and live performance.

In order to strengthen the supervision of HH practice among HCWs, a seasonal feedback and evaluation system was established. Management of infection control department regularly reported HH compliance and HH products consumption in the meeting of Nosocomial Infection Control Management Committee, which included hospital management, department heads, head nurses and focal persons. Also HH compliance and HH products consumption was directly related to the scores of quality control of each department through HH rewards and punishment mechanism. Department with noncompliant HCWs had to pay a fine.

### Part II: Observation of compliance and correctness before and after intervention

#### HH compliance

In our study, a total of 5044 opportunities for HH were recorded in 23 wards before and after intervention. The rate of compliance with HH improved from 66.27% at baseline to 80.53% after intervention (shown in Table 2). After implementing the improvement strategy, doctors had better HH compliance (84.04%) than nurses and other HCWs (81.07% and 69.42%, respectively). The rate of compliance with HH was statistically increased after intervention for each professional category (*P* < 0.05).

**Table 2:** Comparison of HH compliance of HCWs before and after intervention by professional category

	HH compliance before intervention (%)	HH compliance after intervention (%)	χ^2^	P
Doctors	342/459 (74.51)	279/332 (84.04)	10.362	0.001
Nurses	747/1081 (69.10)	2266/2795 (81.07)	64.538	<0.001
Other HCWs	21/135 (15.56)	168/242 (69.42)	100.58	<0.001
Overall	1110/1675 (66.27)	2713/3369 (80.53)	123.99	<0.001

#### HH correctness

A total of 2927 actions with correct HH techniques were recorded. The rate of correctness with HH improved from 47.75% to 88.35% after intervention (shown in Table 3). The increase of correctness applied for all professional categories, which was statistically significant (P < 0.05).

**Table 3:** Comparison of HH correctness of HCWs before and after intervention by professional category

	HH correctness before intervention (%)	HH correctness after intervention (%)	χ^2^	P
Doctors	169/342 (49.42)	201/279 (72.04)	32.669	<0.001
Nurses	349/747 (46.72)	2030/2266 (89.59)	621.30	<0.001
Other HCWs	12/21 (57.14)	166/168 (98.81)	59.123	<0.001
Overall	530/1110 (47.75)	2397/2713 (88.35)	723.76	<0.001

#### HH compliance by indication

The rate of compliance with HH was statistically elevated after intervention for all indications (P < 0.05) except for “after body fluid exposure risk” and “after touching patient surroundings”. The highest relative improvement appeared to be indication “if moving from a contaminated body site to a clean body site during patient care”, from 30.61% to 59.82% (shown in Table 4).

**Table 4:** Comparison of HH compliance before and after intervention of HCWs by indication

	HH compliance before intervention (%)	HH compliance after intervention (%)	χ^2^	P
Before touching a patient	263/489 (53.78)	766/944 (81.14)	119.121	<0.001
After touching a patient	374/489 (76.48)	737/897 (82.16)	6.420	0.011
If moving from a contaminated body site to a clean body site during patient care	15/49 (30.61)	134/224 (59.82)	13.837	<0.001
After body fluid exposure risk	65/84 (77.38)	172/209 (82.30)	0.937	0.333
Before clean/aseptic procedures	160/254 (62.99)	460/552 (83.33)	40.547	<0.001
After touching patient surroundings	150/190 (78.95)	339/415 (81.69)	0.631	0.427
After removing gloves	83/120 (69.17)	105/128 (82.03)	5.589	0.018

## Discussion

Our study identified that implementation of WHO multimodal HH improvement strategy was effective to enhance HH compliance and correctness among HCWs. In the questionnaire survey, over 50% of the participants thought frequently washing hands led to hand skin irritation and dryness, which was a vital cause for noncompliance with HH. In addition, irritated hands might be more vulnerable to be colonized with pathogens [17]. Since cleaning hands frequently is essential for every health care worker, it is important for health care settings to provide proper HH products. Compared with detergent and soap, ABHR has been reported to cause less skin irritation, especially those with emollient properties [18, 19]. The application of skin care products can preserve unimpaired skin, reduce the incidence of skin irritation and dryness and ensure effective hand hygiene [20]. In the last decades, there were concerns that skin care products might pose a negative influence on the efficacy of hand disinfection [21, 22]. With the wide research of well-formulated disinfectants with emollients in recent years, it seems that the efficacy of disinfectants would not be impaired when they are applied with selected, compatible skin care products [23, 24]. In our study, we provided skin care products to our HCWs to minimize the influence on HH compliance due to skin irritation and dryness. Moreover, we encourage our HCWs to use skin care products before work, cleaning and after work under recommendations [25].

Epidemiological evidence have shown that hand contamination of nurses could cause cross-infection in a direct or indirect way, especially in intensive care unit and hemodialysis unit where nurses have many patient contact opportunities [26, 27]. In our study, HH compliance of doctors seemed superior to nurses when stratified by professional category. There were far more opportunities of HH for nurses in most departments than for doctors. Then overcrowding workload of nurses made them provide clinical care to multiple patients without HH to finish their tasks faster. HH compliance in other HCWs was generally lower than doctors and nurses. Poor HH compliance was witnessed among technicians during physiotherapy [28]. Gloves were often used during cleaning work to replace HH by cleaners, which might increase the risk of transmission of bacteria via contaminated gloved hands [29, 30]. Besides, the growing mobility of cleaners and interns made it difficult to accomplish full training on HH. All in all, HH compliance was improved in different professional categories after intervention.

Data of compliance by different HH indication was also investigated in the present study. Compliance with HH improved after intervention across all indications except for “after body fluid exposure” and “after touching patient surroundings” in the observation. We recognized that compliance rates were above 70% for these two indications before intervention, which suggested that our HCWs intended to perform HH when they thought there might be microbial contamination and infection risk. In addition, compliance rates for “before” related indications were promoted after intervention, such as “before touching a patient” and “before clean/aseptic procedures”. These findings revealed that HCWs were inclined to wash their hands to protect themselves rather than protect patients from potential infection, which was noted in previous studies [31, 32]. As for indication “after touching patient surroundings”, HH opportunities with this indication were most commonly associated with lower levels of compliance than following direct patient contact [33]. Traditional Chinese Medicine treatments such as acupuncture and moxibustion are often combined with diathermy machine and herb fumigation device to get better curative effect. Most of our HCWs could perform HH after therapy devices were turned off. But when there was need to adjust the setting of therapy devices, required HH practices were not performed according to our observers. Multiple studies indicated that HAI could be caused by many pathogenic organisms present in the hospital environment and objects frequently touched by patients’ hands, such as bed side rail, door knob, patient record, nurse call button [34, 35]. Moreover, it was of vital importance to strengthen the effectiveness of cleaning and in order to prevent the transmission of pathogens from patient surrounding environment to HCWs and patients [36, 37].

To well acknowledge whether our HCWs mastered the standard handwashing techniques, HH correctness was investigated at the same time. In general, correctness rate was far below our standards before intervention. During the period of investigation, we found two most commonly reasons for low HH correctness rate in our hospital. Most of our HCWs knew the right HH technique procedures but the duration of rubbing hands did not meet our requirement (15 s at least). Inadequate disposable paper towel was another cause for unpleasant HH correctness rate. Therefore our infection control staff took steps to promote correct HH techniques, which included correct HH techniques training, increasing supplies of pocket ABHR and disposable paper towels. As a result, our HCWs’ HH correctness rates were elevated after intervention.

In the last few years, domestic researches on improving HH practice have been reported in succession [38–44]. It is noteworthy that our study is the first observational before-and-after intervention study on improving HH compliance in Xi’an, Shaanxi province. Meanwhile it is the first study implementing WHO multimodal strategy to promote HH practice in a hospital of Traditional Chinese Medicine. HCWs have many patient contact opportunities in the process of Traditional Chinese Medicine treatments such as acupuncture and moxibustion, massage, cupping and other physical therapies. If standard HH practice were not performed, it might increase the risk of cross-infection. Our study summarized the reasons for noncompliance with HH and provided scientific evidence to promote HH practice for other hospitals of Traditional Chinese Medicine. Nevertheless, this study also had certain limitations. The entire observation of HH compliance and correctness were only carried out in general inpatient wards. We planned to observe HH practice in critical departments for infection control management such as emergency, intensive care unit and hemodialysis unit. These places are characterized by high patient volume, critically ill patients and more invasive operations. Improving HH compliance in such places would be meaningful for better infection control. Furthermore, using student nurses as observers might have an impact on observation process. These student nurses observed HH practice of HCWs in clinical wards during their clinical clerkships. Some students told us they recognized their former teaching nurses and classmates in the observation process and thus we concerned that this covert investigation might present the Hawthorn effect. In order to lessen the influence of the Hawthorn effect on HH compliance of HCWs, we plan to train every HCW in our hospital to become a competent observer for HH compliance of their co-workers. In this way every HCW could be our covert observer and we could collect reliable data of HH practice.

## Conclusions

In conclusion, this intervention study has shown that implementation of WHO multimodal improvement strategy could significantly increase compliance and correctness with HH in our hospital. Further investigations with sufficient sample size and larger multicenter series are needed to validate the effectiveness of long-term persistence of HH compliance improvement strategy.

## Additional files

Additional file 1: HH raw data. (SAV 12 kb)

Additional file 2: Possible factors. (SAV 2 kb)

Additional file 3: Questionnaire survey raw data. (SAV 25 kb)

## Abbreviations

ABHR: Alcohol-based hand rub
HAI: Healthcare-associated infection
HCWs: Health care workers
HH: Hand hygiene
TCM: Traditional Chinese Medicine
WHO: World Health Organization
